# Impact of the COVID-19 Pandemic on the Rate of Influenza Vaccination in a Predominately African American Pregnant Population

**DOI:** 10.7759/cureus.30666

**Published:** 2022-10-25

**Authors:** Rachel Shamoun, Patrina Agosta, Sayeh Nabati, Grace D Brannan, Krystine Haglin, Michele Thomas

**Affiliations:** 1 Obstetrics and Gynecology, Ascension Providence Hospital, Southfield, USA; 2 Obstetrics and Gynecology, American University of the Caribbean School of Medicine, Cupecoy, SXM; 3 Research, GDB Research and Statistical Consulting, Athens, USA; 4 Female Pelvic Medicine and Reconstructive Surgery, Indiana University, Carmel, USA

**Keywords:** african american, pregnancy, covid-19 retro, vaccination, influenza

## Abstract

Background

The data is sparse on the uptake of preventative vaccinations during the COVID-19 pandemic in the pregnant population. Our goal was to determine if the COVID-19 pandemic affected the rate of influenza and tetanus, diphtheria, and acellular pertussis (TDAP) vaccination in a predominantly African American pregnant population.

Methods

This retrospective descriptive cross-sectional study compared the influenza vaccination rates of pregnant women 18 years and older between the pre-COVID influenza season (September 1, 2019 to March 1, 2020) and the COVID influenza season (September 1, 2020 to March 1, 2021).

Results

The influenza vaccination rate was statistically significant with a rise from 51.9% pre-pandemic to 72.4% post-pandemic (unadjusted odds ratio (OR) 2.437; 95% confidence interval (CI), 1.64- 3.62; p=0.001). The TDAP vaccination rates remained consistent from the pre-pandemic rate of 65.6% to the pandemic rate of 68.6% (p=0.435).

Conclusion

We concluded that the pandemic had a positive impact on influenza vaccination rates in the pregnant population.

## Introduction

About 10% of the global population is affected by seasonal influenza or flu [1]. The state of pregnancy results in a weakened immune system, making this group of the population particularly vulnerable to severe illness from the flu, sometimes resulting in hospitalization or death of the mother or fetus [2]. Repeat evidence has proven that the risks and complications of influenza are severe enough to warrant effective prevention with the annual influenza vaccine.

Inactivated vaccines such as influenza and tetanus, diphtheria, and acellular pertussis (TDAP) are considered safe and recommended during pregnancy [3-5]. A recent TDAP cohort study involving pregnant and non-pregnant women indicated the vaccine to be safe and well tolerated in both groups [6]. A five-year study involving 8,690 deliveries found influenza vaccination during the first trimester of pregnancy, safe [7].

Flu vaccination additionally provides the benefit of protecting the newborn after birth [2,8]. A case-control study found that in mothers who had the influenza vaccine while pregnant, the vaccine was 92% effective in protecting their infants, six months or less in age, from hospitalization [9]. An additional systematic review found influenza vaccines during pregnancy to be protective against preterm birth and low birth weight [5,10].

Recommendations per the American College of Obstetrics and Gynecology (ACOG) and the Centers for Disease Control and Prevention (CDC) state that all pregnant women should receive their influenza vaccination each year [2,8]. Providers of pregnant women must be vigilant in offering the vaccination as well as counseling their patients about the benefits and risks that coincide with their choice [8]. 

While the benefits of vaccination are well documented, nearly half of women go unvaccinated during pregnancy. According to the CDC, 2018 to 2019 and 2019 to 2020 records have influenza vaccination rates in pregnant women at 53.7% and 61.2%, respectively [11].

Factors affecting vaccination rates are multifactorial, complicated by both lack of patient understanding, lack of provider counseling, and most recently, media attention [11]. For example, the suggestion of a potential association between miscarriage and influenza vaccines following a handful of studies in 2017 became a topic of interest among the media. This led to a decreased influenza vaccine uptake by nearly 15% nationally [12], despite statements from ACOG and the CDC urging vaccination and advocating for its safety [2,8]. This surge of media influence combined with a lack of patient knowledge and poor provider communication led to decreased vaccination rates. 

Racial and ethnic disparities in flu vaccination uptake also exist [13]. Results of the 2012 to 2015 CDC Pregnancy Risk Assessment Monitoring System (PRAMS) data indicated that non-Hispanic Black women had a 30% lower likelihood of receiving a flu vaccine compared to non-Hispanic white women [13]. In another study involving a pregnant population in Northern California, Black women were 1.5 times more likely to be unvaccinated compared to white women [14]. 

A survey study from Israel indicated that during the flu season of 2020 to 2021, which coincided with the second and third wave of COVID-19, there was no change in the uptake of flu vaccination among pregnant and postpartum women [15]. We have not found a similar investigation in the US, particularly in a predominantly African American pregnant population. Through this study, our goal was to determine if the COVID-19 pandemic affected the rate of influenza vaccines in a predominantly African American pregnant population. This article was previously presented as a meeting abstract at the 2022 Ascension Providence 87th Annual Research Day on April 13, 2022.

## Materials and methods

Setting

This study was conducted at the Ascension Providence Academic Obstetrics and Gynecology (OBGYN) Staff Clinic and Partners in Women’s Health in Southfield, Michigan. It was approved by Ascension Providence Hospital’s Institutional Review Board and was considered exempt (approval no.: 1662162-1). The academic obstetrician/gynecologist (OBGYN) staff clinic is associated with the obstetrics and gynecology residency program at Ascension Providence Hospital/Michigan State University College of Human Medicine. At our clinic, every pregnant woman was offered the influenza vaccine as per the standard of care. The TDAP vaccination was also offered if gestational age was appropriate.

Study design and population

This research was conducted as a retrospective descriptive cross-sectional study looking at the vaccination rates during the 2019 to 2020 influenza season (September 1, 2019 to March 1, 2020) considered as the pre-COVID-19 period, and the 2020 to 2021 influenza season (September 1, 2020 to March 1, 2021) as the COVID-19 period. Inclusion criteria included patients with a prenatal appointment in the aforementioned time frame who obtained obstetrical care at Ascension Providence Academic OBGYN staff clinic or Partners in Women’s Health; patients with a documented live, intrauterine pregnancy within the previously mentioned time frame; and patients with two or more prenatal appointments. The exclusion criteria consisted of patients less than 18 years of age. The data were procured by a clinical transformation specialist at the Ascension Medical Group. All patients with two obstetrical appointments within the aforementioned time frame were included and vaccination rates and demographic data were subsequently extracted. 

The 2018 to 2019 and 2019 to 2020 flu vaccination rates in pregnant women were 53.7% and 61.2%, respectively, according to the CDC [11]. The sample size was determined by averaging the percentage of the previous two years to get a percentage equal to 57.5%, with a type I error of 0.05 and a type II error of 0.2. A 10% difference was determined to be clinically significant. The necessary sample size was found to be 280 subjects per group.

All information was obtained using electronic medical records. Patients were identified by the International Classification of Diseases or ICD codes denoting prenatal appointments with care taken to avoid duplicate subjects. Vaccination acceptance and demographic data including age and race were all extracted from electronic databases. The race was reported by the patient on registration forms on the patient’s first visit to that office. 

The primary outcome of this study was the influenza vaccination rates of pregnant women before and during the pandemic. This would be particularly useful in determining how best to counsel patients when encouraging them to receive their influenza vaccination, which is recommended for each pregnant woman per the CDC and the ACOG. Secondary outcomes included whether they received both the TDAP and influenza vaccination or if they permitted one over the other.

Data analysis

We generated descriptive statistics such as frequencies, percentages, and means. A chi-square test was performed to determine the statistical significance of categorical data. The unadjusted odds ratio (OR) was also calculated. For analysis, we used Statistical Package for the Social Sciences (SPSS) version 25.0 (IBM Corp.,, Armonk, NY, USA). Statistical significance was set at a p-value < 0.05.

## Results

For the pre-COVID-19 period from September 1, 2019 to March 1, 2020, 293 women met the study criteria. Of those included, African Americans (84%) made up the majority (Table 1). The average age was 29.4 years in this group. For the post-COVID-19 period from September 1, 2020 to March 1, 2021, 185 women met the study criteria. Of those included in the study, most of the women were African American (80%) and white (12%) (Table1). The average age was 29.1 years in this group. 

**Table 1:** Patients' race rounded to the nearest tenth of a percent

Race	Pre-COVID-19		During COVID-19	
	Frequency	%	Frequency	%
Black or African American	246	83.96	148	80.00
White	27	9.22	23	12.43
Black and white	2	0.68	0	0.00
Asian	3	1.02	2	1.08
American Indian or Alaska Native	1	0.34	2	1.08
Other race	3	1.02	2	1.08
Declined to report race	11	3.75	8	4.32
Total	293	100	185	100

Comparing pre-COVID-19 to COVID-19 periods, the influenza vaccination rate rose from 51.9% to 72.4% for flu (unadjusted OR, 2.437 (95% confidence interval (CI), 1.64- 3.62), (p=0.001). The TDAP vaccination rates remained consistent with pre-pandemic rate of 65.6% to post-pandemic rate of 68.6% (unadjusted OR, 1.169 (95% CI, .79- 1.73), (p=0.435). Rates for having both influenza and TDAP vaccine were not statistically significant (unadjusted OR, 1.090 95% CI, .75- 1.58), (p=0.652), pre-covid (40.6%) and during covid (42.7%). 

## Discussion

Our research revealed that there was a statistically significant increase in influenza vaccinations in the pregnant population during COVID-19 as compared to pre-COVID-19. While we did not see an increase, it was encouraging to see that TDAP vaccination rates remained the same during the pandemic.

Overall, our experience was that during the pandemic increased awareness, inquisition by patients, and counseling by healthcare providers played a key role. Throughout the COVID-19 pandemic, the CDC and other government agencies along with the media were consistently updating the public, making viruses at the forefront of people’s minds. With a surplus of information readily available, we believe patients were more likely to think, talk, and enquire about other viruses. Related to this, one study found that in addition to their physician’s recommendation, CDC was a top source for pregnant women on influenza and TDAP vaccination information [16].

Increased awareness starts with patients, but much of this burden is placed on healthcare providers as well. A study reported that when providers specifically recommended the influenza vaccine, patients were more agreeable [17]. However, less than half of the women surveyed in another study reported receiving a recommendation from their physician [18]. A more recent survey of multi-ethnic pregnant women in Italy found an increased flu vaccine acceptance during COVID-19 compared to pre-COVID-19 [19]. They also observed that patients were more accepting of the flu vaccine if recommended by their provider. The increased awareness from providers during COVID-19 may have also led to increased counseling and education, as well as stronger recommendations by healthcare providers for the influenza vaccine. 

The presentation of comprehensible material on complex topics becomes relevant for healthcare providers to keep in mind. Complex topics and medical terminology often prove to be difficult to discuss in layman's terms. In order to help, the CDC website provides patient-friendly and useful pamphlets and information sheets on vaccine safety and benefits specifically relating to pregnant women [20]. Thus, effective and understandable counseling and resources likely impact patient vaccination rates. 

Of greater importance, our population was more than 80% Black or African American. Racial disparities in the healthcare system are evident in other preventative efforts, as seen in another study showing that Black patients had the lowest prevalence in completing the HPV vaccination series, at a 28.7% completion rate [21]. We believe the statistically significant increase in influenza vaccination rate from 2020 to 2021 to hold even more weight, as it represents a change in such a vulnerable community. 

Study limitations

A limitation of our study is that it involved a small patient population in one geographical area of the United States. Further studies with enlarged sample sizes may be helpful to apply this result to the generalized population. Another limitation is the varying levels of counseling on the importance of vaccinations in pregnant women provided by each provider. Our study did not evaluate how patients were counseled and different counseling styles provided to patients may affect the vaccination rates. 

## Conclusions

Overall, our study provided an optimistic view of a predominantly African American pregnant patient population’s willingness for influenza vaccination during the COVID-19 pandemic, and it provides insight for potentially sustaining this increased rate in this vulnerable population even after the pandemic concludes. Multiple components, with the COVID-19 pandemic itself largely being a contributing factor, played a part in the increase of influenza vaccination among our patient population. Further studies should include the rate of COVID vaccination and its correlation to the influenza vaccination rates. 
